# Mini-GRID enhances survival and reduces toxicity in an orthotopic murine model of oral squamous cell carcinoma: A proof-of-concept study

**DOI:** 10.1016/j.ctro.2025.101101

**Published:** 2025-12-29

**Authors:** Loris Roncali, Maria Isabel Acuña-Perez, Julie Espenon, Manuel Sánchez-García, Victor Luna-Vega, Eva G. Kölmel, Cristèle Gilbert, Mathieu Sertorio, Marjorie Juchaux, Yolanda Prezado

**Affiliations:** a New Approaches in Radiotherapy Lab, Center for Research in Molecular Medicine and Chronic Diseases (CiMUS), Instituto de Investigación Sanitaria de Santiago de Compostela (IDIS), University of Santiago de Compostela, E-15782 Santiago de Compostela, Spain; b Institut Curie, PSL University, CNRS MR3347, Inserm U1021, Radiobiology and Cancer Signaling, F-91400 Orsay, France; c Paris-Saclay University, CNRS UMR3347, Inserm U1021, Radiobiology and Cancer Signaling, F-91400 Orsay, France; d Department of Radiophysics and Radiological Protection, University Hospital of Santiago de Compostela, E-15782 Santiago de Compostela, Spain; e Department of Radiation Oncology, University of Cincinnati College of Medicine, Cincinnati, OH 45221, USA; f University of Cincinnati Cancer Center, University of Cincinnati, Cincinnati, OH 45221, USA; g Oportunius Program, Galician Agency of Innovation (GAIN), Xunta de Galicia, E-15781 Santiago de Compostela, Spain

**Keywords:** Spatially fractionated radiation therapy, Mini-GRID radiation therapy, Oral squamous cell carcinoma, Orthotopic tumour model

## Abstract

**Background and purpose:**

Oral squamous cell carcinoma (OSCC) remains a major clinical challenge, with limited therapeutic options and poor outcomes. Among the emerging approaches to address this unmet need, spatially fractionated radiotherapy (SFRT) using mini-GRID patterns has emerged as a promising approach, delivering high radiation doses through narrowly collimated beamlets that spare intervening normal tissue and may enhance the therapeutic index. This study evaluated the efficacy and safety of mini-GRID RT in an orthotopic, syngeneic murine model of OSCC.

**Materials and methods:**

C57BL/6 mice bearing MOC1 tumors were randomized to receive conventional radiotherapy (conv-RT; 20 Gy), mini-GRID RT (20 Gy average dose), or no treatment. Endpoints included survival, tumor growth, body weight, and histopathological assessment of tumor and surrounding normal tissues.

**Results:**

Mini-GRID RT significantly extended median survival (56 days) compared with controls (36 days; *p* = 0.0456) and conv-RT (35 days; *p* = 0.0181). Tumor growth was delayed by approximately 20 days in the mini-GRID group, with long-term survivors regaining baseline body weight within 15 days post-irradiation. Fewer animals in the mini-GRID group required early euthanasia due to acute toxicity compared with the conv-RT group. Histological examination revealed no significant increase in normal tissue damage in mini-GRID-treated mice relative to conv-RT.

**Conclusion:**

These findings indicate that mini-GRID RT can improve survival while maintaining a favorable toxicity profile in OSCC, supporting its potential as a novel high-dose radiotherapy approach. Further studies are warranted to elucidate underlying mechanisms, including vascular and immune-mediated responses, and to assess its translational relevance in clinical practice.

## Highlights

• Mini-GRID RT extended survival compared with control and conv-RT.
• Normal tissues were preserved under mini-GRID, with reduced acute toxicity.
• Toxicity increased at 25 Gy, indicating 20 Gy as the ideal single fraction.

## Introduction

Oral squamous cell carcinoma (OSCC) is a common subtype of head and neck squamous cell carcinoma (HNSCC), arising from the oral epithelium. SCC accounts for 90 % of head and neck cancers (HNCs), which include tumors of the oral cavity, pharynx, and larynx [1]. Its impact is profound: OSCC impairs appearance, speech, swallowing and taste, and leads to substantial morbidity and mortality. Despite therapeutic advances, 5-year overall survival remains 70–90 % for stage I-II OSCC but falls to 30 % in advanced disease [2]. Conventional treatments – surgery, radiotherapy (RT), and chemotherapy, alone or combined – cause significant morbidity and are often ineffective in advanced stages [3].

RT remains a cornerstone of HNC treatment, but curative doses are limited by toxicities such as mucositis, often leading to treatment interruptions [4,5]. While fractionation strategies have been explored, toxicity remains a major challenge. Traditionally, conventional RT (conv-RT) aims to deliver homogeneous doses across the planning target volume to eradicate clonogenic tumor cells and avoid underdosing. Although established techniques such as the simultaneous integrated boost (SIB) allow differential dose delivery within a single treatment course, dose escalation remains constrained by conventional fractionation and organ-at-risk tolerance, resulting in largely biologically homogeneous dose distributions [6]. However, with hypofractionated ablative RT, intratumoral dose heterogeneity is increasingly recognized as advantageous [7].

Among emerging strategies, spatially fractionated radiotherapy (SFRT) has become one of the most promising candidates to improve the therapeutic index in cancer. It delivers radiation in alternating high- and low-dose regions, referred to as peaks and valleys, thereby allowing escalation of tumor dose while limiting toxicity to surrounding normal tissues [8]. SFRT has demonstrated potential in the management of bulky tumors, with evidence of reduced normal tissue toxicity in gliomas, lung cancers, and other malignancies [9], alongside effective activation of the immune response [[10], [11], [12]] and induction of immune memory [11].

Four main SFRT modalities exist: GRID RT [13], lattice RT (LRT) [14] (both with cm-scale beams), microbeam RT (MRT) [15], and minibeam RT (MBRT) [16] (both with submillimeter beams). GRID [13,[17], [18], [19], [20], [21], [22]] and lattice RT [23,24] have been used clinically, mainly for palliation, whereas MRT and MBRT remain largely preclinical, though early applications are encouraging [25]. Smaller beam sizes exploit dose-volume effects [26], enhancing normal tissue tolerance and enabling safe dose escalation in resistant tumors, with curative potential for otherwise untreatable malignancies such as gliomas [[27], [28], [29], [30]].

Conventional GRID and LRT are limited by lateral scattering from megavoltage LINAC beams, which increases valley dose and reduces tissue sparing. Both also require relatively large beam sizes (∼1 cm^2^) to maintain adequate dose rates in GRID, or to achieve sufficient precision with the multileaf collimator in LRT. Mini-GRID RT, a novel SFRT adaptation, refines the GRID concept using 1–2 mm beams – around 5–8 times narrower than with conventional GRID [31] – enabling to further exploit the dose-volume effects (the narrower the beam, the higher the tolerance of normal tissues [26]). Implemented on a TrueBeam® 6XFFF LINAC (Varian, Palo Alto, USA) at the University Hospital of Santiago de Compostela [32], mini-GRID has shown improved safety over conv-RT in preclinical brain irradiation, as evidenced by imaging analyses, behavioral testing, and histopathological assessments [33]. Moreover, Monte Carlo simulations indicated reduced doses to organs at risk in the context of preoperative breast cancer [34].

Collectively, these findings highlight mini-GRID RT as a promising OSCC therapy. Notably, local recurrences occur in nearly 50 % of human papillomavirus (HPV)-positive cases despite completed RT, and re-irradiation is often precluded by toxicity. Mini-GRID RT could reduce recurrence risk and may also enable safer re-irradiation in otherwise untreatable scenarios. Here, we investigated mini-GRID RT in an orthotopic HPV-OSCC murine model. We hypothesized that mini-GRID RT would improve survival and reduce morbidity compared with conv-RT, focusing on survival outcomes and preliminary histological assessment of adjacent normal tissues.

## Materials and methods

### Ethical statement

All procedures were approved by the Bioethics Committee of the University of Santiago de Compostela (Code: 15012/2024/014) and conducted in accordance with Spanish (RD 53/2013) and EU (2010/63/EU) regulations. Experiments followed the ARRIVE guidelines, with measures implemented to minimize discomfort and pain. Animals were housed at the Center for Experimental Biomedicine of Galicia (CEBEGA, Santiago de Compostela, Spain).

### Cell line and culture conditions

MOC1 mouse oral carcinoma cells (Kerafast: EWL001-FP) were detached using 0.05 % trypsin (25300-062, Gibco) for 30 s, followed by 0.25 % trypsin (25200-072, Gibco) for 5–8 min at 37 °C, and resuspended in “IMDM MOC line medium”. This medium consisted of 62.6 % Iscove's Modified Dulbecco's Medium (IMDM) 1X (12440-046, Gibco), 31.3 % Ham’s F-12 nutrient mixture 1X (117656–054, Gibco), 5 % fetal bovine serum (21523002, Corning), 1 % penicillin/streptomycin (15140-122, Gibco), 5 mg/mL insulin (I6634, Sigma-Aldrich), 0.04 μg/mL hydrocortisone (H0135, Sigma-Aldrich), and 5 ng/mL EGF (01-107, Merck Millipore). Medium was filtrated in a 1L vacuum filter/storage bottle system, 0.22 µm pore 54.5 cm^2^ PES membrane (431098, Corning). Cells were centrifuged at 120 *g,* counted, and cultured in a humidified incubator (37 °C, 21 % O_2_, 5 % CO_2_). Experiments were conducted when cultures reached 80 % confluence. Routine mycoplasma testing was performed.

### Animal model

Thirty-three 8-weeks male C57BL/6 mice were purchased from the Center for Experimental Biomedicine of Galicia (CEBEGA, University of Santiago de Compostela, Spain) and acclimated for one week before experimentation. Animals were housed in groups of 4–5 per polycarbonate cage under controlled conditions (21.1 °C ± 1 °C, 50 ± 5 % humidity, 12-h light/dark cycle) with *ad libitum* access to tap water and food. Male mice were selected to minimize variability in radiation response associated with female hormonal fluctuations, particularly progesterone [35]. In addition, OSCC disproportionately affects men and is closely associated with exposure to tobacco, alcohol, betel nut and HPV [36]. Each mouse was considered an independent experimental unit.

### Orthotopic tumor grafting

MOC1 cells were detached using trypsin, counted, and assessed for viability by trypan blue exclusion. The injection suspension contained 1.5 million MOC1 cells in 50 µL PBS mixed with 50 µL Matrigel (354234, Corning). Analgesia was provided with buprenorphine (5 μg/g, intraperitoneal) 10 min before grafting and bupivacaine (0.42 μg/g, subcutaneous) to the contralateral cheek 2 min before. Anesthesia was induced with 4 % isoflurane (Isoflutek, 1000 mg/g) and maintained under 2.5 %. Mice were injected at the right oral commissure with 100 µL of MOC1 suspension (*n* = 33), following the protocol of Dr. Mathieu Sertorio (Department of Radiation Oncology, The Barrett Cancer Center, Cincinnati, OH, USA). Tumor engraftment was 96.97 %. Of 33 inoculated mice, 20 (60.6 %) developed measurable tumors at the treatment initiation (day 26) and were included in the main study. Five additional mice were included to assess healthy tissue toxicity and were assigned to a mini-GRID RT dose-escalation cohort (25 Gy), regardless of tumor size. These animals were excluded from survival analysis.

### Irradiations

Irradiations were performed with a 6 MV flattening-filter-free LINAC (TrueBeam®, Varian) at the University Hospital of Santiago de Compostela. Mini-GRID irradiations followed previously described procedures [33]. A 1-cm bolus was applied. Beam width at the tumor site measured 1.3 mm, with a center-to-center spacing of 3.6 mm. Treatments were delivered 26 days after tumor inoculation. To make sure that tumors received more than one peak dose, only mice with tumors larger than 6 mm in diameter (measured perpendicular to the irradiation axis) were included in the study (mean volume: 237.6 ± 116.1 mm^3^). Animals were assigned to three groups for the survival study: untreated controls (*n* = 6), conv-RT 20 Gy (*n* = 7), or mini-GRID RT 20 Gy (*n* = 7), and one dose escalation group receiving mini-GRID RT at 25 Gy (*n* = 5). The conv-RT group received a single 20 Gy dose to a 2 × 2 cm field without modulation. Mini-GRID RT was delivered at an average of either 20 ± 1 Gy (peak 35 ± 3 Gy; valley 5.0 ± 0.04 Gy; peak-to-valley dose ratio PVDR = 7) or 25 Gy (*n* = 5; peak 44 ± 3 Gy, valley 6.25 ± 0.5 Gy; PVDR = 7.04). Gafchromic films were positioned at the bolus entrance for irradiation quality control.

### Survival and toxicity assessment

Mice were weighed and tumor volumes were measured at least 5 times per week. The longest longitudinal (length) and transverse (width) diameters were measured with a digital calliper, and volumes calculated as: length × width^2^ × 0.5 [37]. Oral mucositis was assessed daily for 15 days post-irradiation by serial macroscopic examination of the oral cavity using a standardized grading scale based on erythema, ulceration, and mucosal integrity. Hydration status was monitored daily over the same period through body weight measurements and systematic clinical assessment of dehydration-related signs, including reduced skin turgor, sunken eyes, dry oral mucosa, decreased grooming and activity, and overall deterioration of general condition. Subcutaneous hydration and soft diet were provided as needed to minimize weight loss. Humane endpoints included body weight loss >20 % of initial weight, tumor volume >600 mm^3^, combined with deterioration of general condition with persistent pain symptoms unrelievable by analgesics (*e.g.*, reduced activity, reduced food/water intake, or other signs according to the Mouse Grimace Scale [38]).

### Immunohistochemistry and histological analysis

At the survival study endpoint, tumors were harvested from all animals (*n* = 25). In a subset of each group, lip skin (control *n* = 4; conv-RT 20 Gy *n* = 4; mini-GRID 20 Gy *n* = 5; mini-GRID 25 Gy *n* = 4), tongue (control *n* = 4; conv-RT 20 Gy *n* = 4; mini-GRID 20 Gy *n* = 7; mini-GRID 25 Gy *n* = 4), and submandibular salivary glands (control *n* = 4; conv-RT 20 Gy *n* = 4; mini-GRID 20 Gy *n* = 7; mini-GRID 25 Gy *n* = 4) were also collected. Toxicity analyses were performed on a subset of animals, restricted to tissue specimens meeting predefined quality criteria, namely adequate structural integrity and orientation, and absence of major processing-related artifacts such as tears or tissue distortion. Samples not fulfilling these criteria were excluded to prevent biased interpretation of histopathological endpoints. Tissues were fixed in zinc-formalin (Z2902, Sigma-Aldrich), washed with PBS, dehydrated in ethanol, and paraffin-embedded. 4-µm sections were prepared. For hematoxylin and eosin (H&E) staining, sections were deparaffinized (60 °C, 1 h; xylene 10–15 min), rehydrated through graded ethanol (100 %, 96 %, 70 %, 50 %, 5 min each), and rinsed in distilled water. Hematoxylin staining was performed for 10 min, followed by eosin counterstaining for 5 min. Slides were then dehydrated (70 %, 96 %, 100 %; xylene) and mounted. For immunohistochemistry, sections were deparaffinized and subjected to antigen retrieval with Tris-EDTA buffer in a PT Link system (Agilent, 20 min). Endogenous peroxidase was blocked with 3 % H_2_O_2_ (10 min), followed by PBS washes. Sections were incubated overnight at 4 °C with primary antibodies against CD68 (28058–1-AP, Fisher Scientific) or vimentin (ab92547, Abcam) according to manufacturer’s instructions. After PBS washing, secondary antibody (SM802, Dako) was applied for 30 min at room temperature. Signal was detected with DAB (K3468, Dako, 1 min) and counterstained with hematoxylin (SM806, Dako, 10 min). Finally, sections were dehydrated and mounted for analysis on a Nanozoomzer S20 digital slide scanner (Hamamatsu).

### Statistical analysis

Group allocation was stratified by tumor size to control for this confounding factor. Survival was analyzed using Kaplan-Meier curves, with significance assessed by Log-Rank test (*p* < 0.05). Hazard ratios (HR) and 95 % confidence intervals (CI) were estimated using Cox proportional hazards regression, with weight included as a covariate. Sample sizes were determined by feasibility, as this exploratory study aimed to generate hypotheses and identify response patterns not previously described. Data were analyzed with GraphPad Prism v10.4.2.

## Results

### Mini-GRID therapy enhances survival in an orthotopic OSCC model after a 20 Gy average dose

**Fig. 1:**
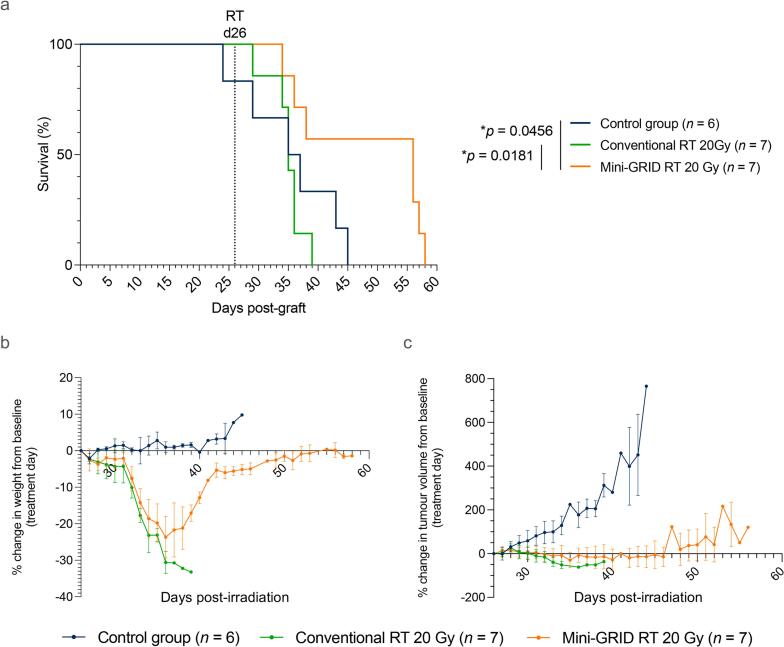
**(a)** Kaplan-Meier survival curves of C57BL/6 mice following orthotopic injection of 1.5 × 10^6^ MOC1 cells at the oral commissure. 26 days post-tumor graft, mice were treated with conv-RT (20 Gy) or mini-GRID RT (average dose of 20 Gy). Statistical significance was determined using the Log-Rank test. *p < 0.05. **(b)** Change in body weight relative to the treatment day. **(c)** Change in tumor volume relative to the treatment day.

Kaplan-Meier analysis demonstrated significantly prolonged survival in the mini-GRID group (Fig. 1a, Table S1). Median survival was 36 days in controls (*n* = 6), 35 days with conv-RT (20 Gy; *n* = 7), and 56 days with mini-GRID RT (20 Gy; *n* = 7). Mini-GRID RT significantly improved survival compared with both control (*p* = 0.0456, HR = 0.24, 95 % CI: 0.06–0.97) and conv-RT (*p* = 0.0181, HR = 0.18, 95 % CI: 0.05–0.75). On average, this translated into a ∼3-week delay in tumor- or toxicity-related euthanasia relative to conv-RT.

All irradiated mice developed mild oral mucositis and reduced food intake, resulting in body weight loss between days 7 and 15 post-irradiation. By day 8, mean body weight change relative to baseline was −21.1 % in conv-RT, −18.8 % in mini-GRID RT, and +1.4 % in controls (Fig. 1b). Hydration and a soft diet stabilized weight in 5/7 mini-GRID-treated animals, compared with only 1/7 in conv-RT. In surviving mini-GRID mice, weight returned to baseline ∼15 days after irradiation, whereas control mice maintained stable weight until tumor burden reached experimental endpoint (Fig. S1a). In the mini-GRID group, 4/7 mice eventually reached tumor volume endpoint, while 5/7 conv-RT animals required early euthanasia for pain, consistent with greater local toxicity (Fig. S1b, Table 1). Both conv-RT and mini-GRID RT delayed tumor growth (Fig. 1c), but mini-GRID RT achieved more durable tumor control, with regrowth delayed by ∼20 days. Conv-RT reduced tumor growth initially but was offset by toxicity-driven mortality (Fig. S1b).

**Table 1:** **Summary of the survival study.** Data from two independent experiments.

Protocol	n	Surviving mice	Median survival (days)	Signs of pain	*Cause of sacrifice*		
					Weight loss > 20 % highest weight	Tumor size > 600 mm^3^	Pain
Control group	6	0	36	0	0	6	0
Conv-RT 20 Gy	7	0	35	6	1	1	5
Mini-GRID RT 20 Gy	7	0	56	3	1	4	2

In an exploratory toxicity study, a subset of mice with smaller tumors (*n* = 5) received mini-GRID RT at 25 Gy. This cohort was excluded from survival analyses. 3/5 mice required euthanasia due to >20 % weight loss and pain, suggesting higher toxicity than with 20 Gy (Fig. S2, Table S2).

### Tumor necrosis and normal-tissue preservation after mini-GRID RT

**Fig. 2:**
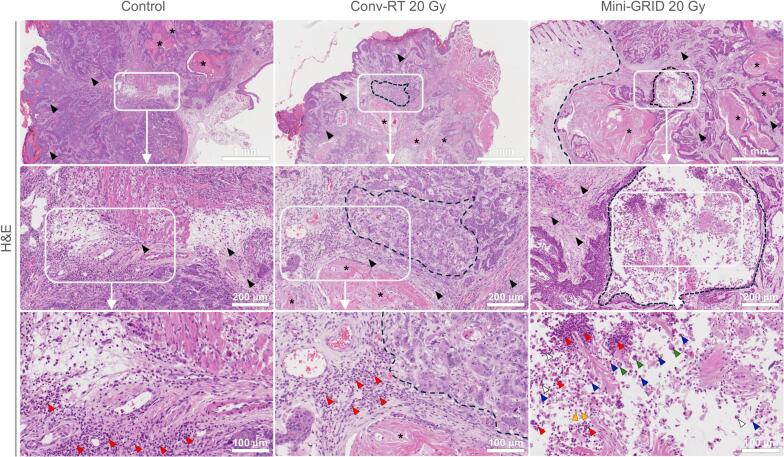
**Histological evaluation of tumors at endpoint after mini-GRID RT.** Tumors from the survival study groups (control *n* = 6; conv-RT 20 Gy *n* = 7; mini-GRID RT 20 Gy *n* = 6) were harvested at the endpoint and processed for H&E staining for histological evaluation. Black arrowhead: desmoplasia; asterisk: keratinization; black dashed area: necrosis; red arrowhead: lymphocyte; blue arrowhead: macrophage; yellow arrowhead: neutrophil; white arrowhead; eosinophil; green arrowhead: giant cell. (For interpretation of the references to colour in this figure legend, the reader is referred to the web version of this article.)

To compare the effects of mini-GRID RT and conv-RT, we conducted histological analysis of tumor tissues from the survival study. Control tumors exhibited viable architecture on H&E staining, consisting of densely nucleated tumor cells with basophilic cytoplasm. Hallmarks of squamous cell carcinoma, including keratinization and dense fibrous stroma (desmoplasia), were evident. No necrotic regions were observed, indicating active growth. In contrast, tumors treated with conv-RT at 20 Gy showed extensive coagulative necrosis with eosinophilic amorphous areas, lysed nuclei, and a complete loss of tissue architecture. Tumors treated with mini-GRID at 20 Gy displayed foci of complete necrosis similar to conv-RT, interspersed with preserved tumor areas. In conv-RT-treated tumors, enhanced vascularization primarily supported a diffuse infiltration of lymphocytes. In contrast, mini-GRID-treated tumors exhibited a more heterogeneous immune response, characterized by the presence of lymphocytes, neutrophils, eosinophils, and macrophages, which tended to cluster within necrotic areas (Fig. 2).

**Fig. 3:**
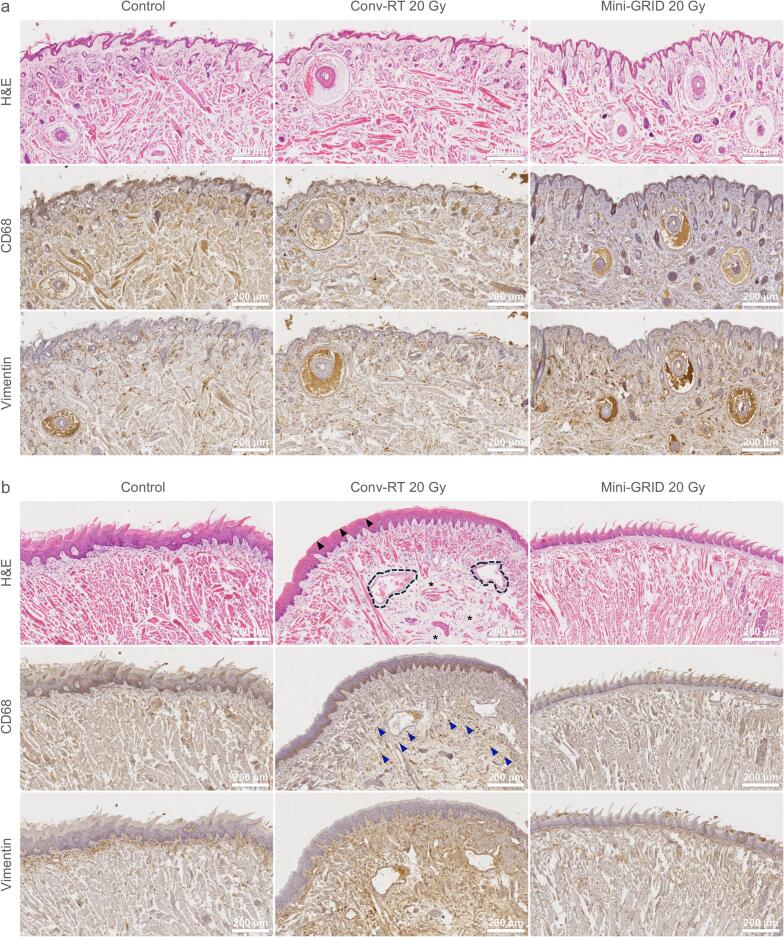
**Histological analysis of lip and tongue tissues following radiation therapy.** H&E staining, as well as CD68 and vimentin immunostaining, were performed on sections of **(a)** lip skin and **(b)** tongue collected from a subset of mice in the survival study. For lip skin samples: control (*n* = 4), conv-RT 20 Gy (*n* = 4), and mini-GRID RT 20 Gy (*n* = 5). For tongue samples: control (*n* = 4), conv-RT 20 Gy (*n* = 4), and mini-GRID RT 20 Gy (*n* = 7). Black arrowhead: loss of filiform papillae; black dashed area: increased blood vessel size; blue arrowhead: monocyte/macrophage infiltration. (For interpretation of the references to colour in this figure legend, the reader is referred to the web version of this article.)

A preliminary toxicity assessment was performed on healthy tissues from a subset of mice (refer to the Methods section for group sizes). In addition to H&E, CD68 immunohistochemistry was used to assess macrophage infiltration, and vimentin staining identified mesenchymal cells, particularly activated fibroblasts, to evaluate post-radiation fibrosis [39]. In the lower lip skin, conv-RT induced no apparent tissue alterations, and CD68 and vimentin staining remained negative. Mini-GRID RT preserved epidermal architecture and thickness, and showed low but positive CD68 and vimentin expression, consistent with mild inflammation in the absence of histologically detectable fibrosis (Fig. 3a).

In tongue tissue, conv-RT led to the loss of filiform papillae and dilation of blood vessels, along with advanced fibrosis characterized by atrophic squamous epithelium and collagenization of the subepithelial tissue with scant inflammatory cells. CD68 staining confirmed monocyte/macrophage infiltration, while increased vimentin expression supported the presence of fibrosis. In contrast, mini-GRID RT preserved normal lingual architecture, with no significant inflammatory response. CD68 and vimentin expression remained at control levels, indicating the absence of fibrosis (Fig. 3b).

**Fig. 4:**
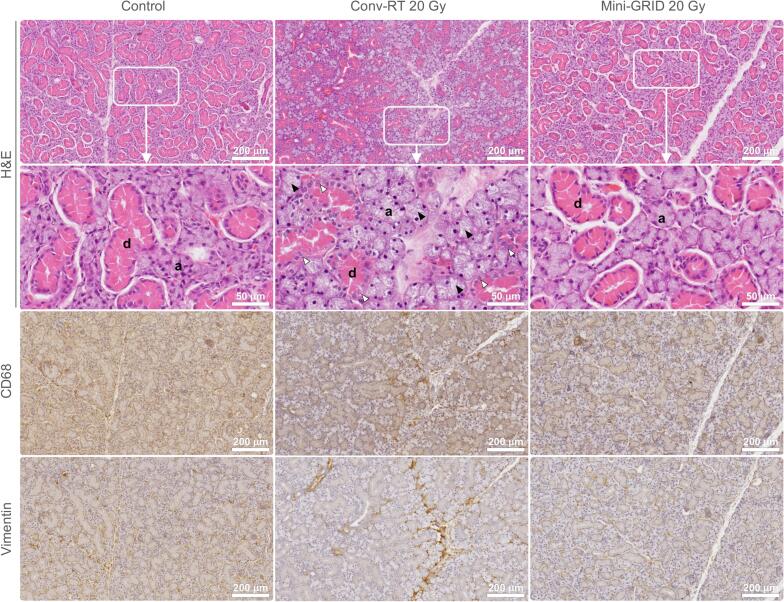
**Histological analysis of salivary gland tissues following radiation therapy.** H&E staining, along with CD68 and vimentin immunostaining, were performed on sections of submandibular salivary glands collected from a subset of mice in the survival study. Sample groups: control (*n* = 4), conv-RT 20 Gy (*n* = 4), and mini-GRID RT 20 Gy (*n* = 7). d: execretory duct; a: serous acinus; black arrowhead: degranulation of serous acini; white arrowhead: karyolysis.

In submandibular salivary glands, conv-RT caused marked tissue injury, characterized by acinar degranulation, nuclear karyolysis, and disruption of parenchymal organization. In contrast, these lesions were absent following mini-GRID RT, where the acinar and ductal architecture was largely preserved and cellular damage was minimal. CD68 and vimentin staining levels were comparable to controls in mini-GRID-treated tissues, with no significant differences observed between groups (Fig. 4).

In tumors treated with mini-GRID RT 25 Gy, histological analysis revealed more pronounced tissue damage in the lip and tongue, characterized by advanced fibrosis and marked inflammatory cell infiltration, as confirmed by CD68 staining. Vimentin expression was also elevated in these tissues, consistent with the observed degree of fibrosis. In contrast, both CD68 and vimentin staining remained low in the salivary glands, where overall tissue architecture was largely preserved (Fig. S3).

## Discussion

This proof-of-concept study demonstrates that a single average dose of 20 Gy of mini-GRID RT improves survival in an orthotopic, syngeneic OSCC model while maintaining a favorable toxicity profile compared with equivalent dose of 20 Gy in conv-RT. In these conditions, mini-GRID RT extended median survival, delayed tumor growth, and reduced morbidity, as indicated by fewer euthanasia due to pain or feeding impairment (Table 1). These results are consistent with previous long-term studies in healthy rat brain showing sustained tissue preservation and minimal late effects after mini-GRID irradiation [33]. This finding is particularly relevant for head and neck cancers, where mucositis, dysphagia, xerostomia, and pain remain major dose-limiting toxicities that often lead to treatment interruptions and worsen clinical outcomes [4,5].

Our data align with the broader literature on spatially fractionated radiotherapy (SFRT), in which techniques such as microbeam (MRT), minibeam (MBRT), and mini-GRID RT exploit dose-volume effects to enhance normal tissue tolerance [9]. Preclinical MBRT studies have consistently shown long-term preservation of healthy tissues [16,28,[40], [41], [42], [43]] and comparable or improved tumor control relative to uniform irradiation [29,30,44,45]. In particular, comparison with a recent MBRT valley-dose study performed by our group, provides further context [46]. In this work, we treated healthy murine oral cavity with 0.5 mm minibeams spaced 1.1 mm and compared conventional 16 Gy or 20 Gy irradiation with several MBRT peak:valley combinations. Conv-RT and the MBRT groups receiving high valley doses (16 Gy) reached toxicity endpoints within 9–11 days, whereas MBRT schemes with lower valley doses did not.

In the present study, despite the use of much thicker beams (1.3 mm-wide at the tumor site), the histopathological analyses confirmed that mini-GRID RT preserved epithelial and glandular structures, while conv-RT caused classical radiation-induced lesions such as acinar degranulation and architectural disorganization (Fig. 3) [39,47,48].

Although both 20 Gy regimens initially slowed tumor growth, the survival benefit of mini-GRID RT appears to be mainly attributable to its lower acute toxicity rather than to durable local tumor control, since all tumors eventually progressed. The exploratory 25 Gy cohort suggested a threshold of tolerability: several mice reached humane endpoints for weight loss and pain despite evident tumor cytoreduction (Fig. S2, Table S2). Histological evaluation partially explained this higher toxicity, as increased vimentin expression in lip, tongue, and salivary glands indicated enhanced fibrotic activity (Fig. S3). Therefore, 20 Gy likely represents the single-fraction maximum tolerated dose in this model. Our study is limited here by the investigation of only one dose of 20 Gy, therefore further studies should also explore lower doses to check if this superior efficacy is preserved. Future optimization should refine beam geometry and investigate multi-fraction or cross-fire configurations to widen the therapeutic window. Notably, Grams *et al.* have demonstrated that multi-angle MBRT can minimize peak overlap while preserving spatial modulation [25], offering a relevant strategy for mini-GRID RT development.

Mechanistically, mini-GRID RT probably shares biological principles with other submillimeter SFRT modalities. Proposed mechanisms include vascular modulation [49], intercellular signaling and bystander effects [50], immune activation [51], preservation of normal stem-cell niches [52], and diffusion of reactive oxygen species across dose gradients [53]. While these processes were not directly assessed here, our histological data suggest that high-dose peaks may induce vascular collapse and coagulative necrosis, whereas low-dose valleys preserve viable stromal and immune cells that could facilitate repair and immune activation. This spatial synergy might generate an immune-supportive microenvironment that contributes to the observed survival extension and delayed tumor regrowth.

Given that the MOC1 line is relatively immunogenic [54,55], immune-mediated mechanisms may have been amplified in this model. Future studies should therefore include less immunogenic OSCC models (*e.g.*, MOC2) to assess the relative contribution of immune versus non-immune effects. Moreover, longitudinal analyses with larger cohorts are needed to capture the temporal evolution of inflammation, fibrosis, and vascular remodeling. Mechanistic studies employing multiplex immunohistochemistry, flow cytometry, and single-cell transcriptomics could help delineate immune and vascular pathways involved, focusing on CD8^+^/Treg balance, dendritic activation, cytokine profiles, and endothelial integrity.

## Conclusions

In summary, a 20 Gy average dose of mini-GRID RT improved the therapeutic ratio in an orthotopic OSCC model by reducing acute morbidity without increasing normal-tissue injury, while achieving meaningful tumor growth delay. Together with early clinical MBRT data demonstrating feasibility and symptomatic benefit [25], and recent evidence supporting the implementation of submillimeter SFRT on standard LINACs [32,33], these findings support the continuation of exploration of mini-GRID RT protocols, to demonstrate its increasing translational potential. Future work should focus on (i) exploration and comparison of different doses schemes, (ii) optimization of spatial and temporal parameters, (iii) elucidation of the underlying immune and vascular mechanisms, and (iv) combination strategies with immunotherapy – such as PD-1/PD-L1 blockade – to exploit the potential for immune priming and improve outcomes in locally advanced or recurrent head and neck cancers.

## Declaration of competing interest

The authors declare that they have no known competing financial interests or personal relationships that could have appeared to influence the work reported in this paper.
