# Supplementary material for: Mini-GRID enhances survival and reduces toxicity in an orthotopic murine model of oral squamous cell carcinoma: A proof-of-concept study
Source: Clin Transl Radiat Oncol. 2025 Dec 29;57:101101. doi: 10.1016/j.ctro.2025.101101 (PMC12804384; doi:10.1016/j.ctro.2025.101101)
Supplement: Supplementary Data 1 [file mmc1.pdf]

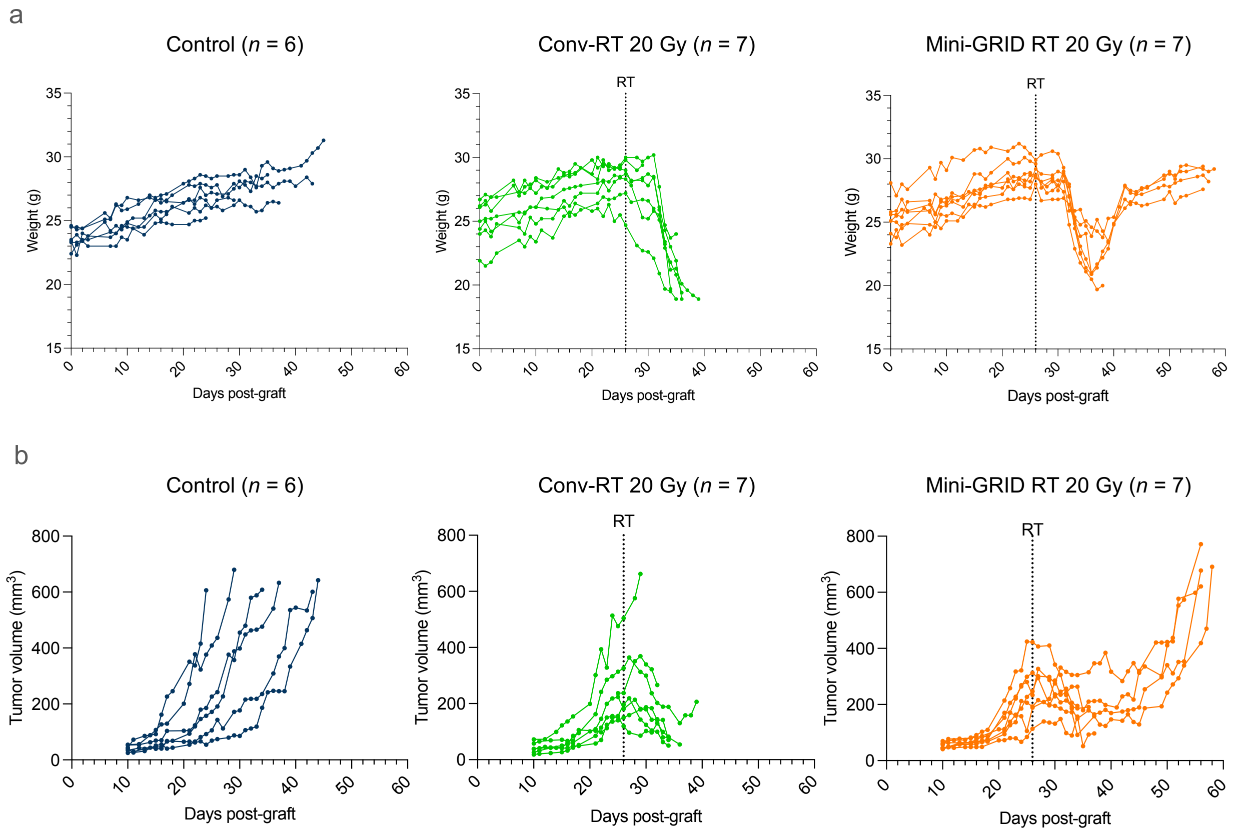


**Figure S1. Individual mouse monitoring during the survival study. (a)** Individual body weight curves for mice in the control, conv-RT (20 Gy), and mini-GRID RT (20 Gy) groups. **(b)** Individual tumor volume curves for all experimental groups. Individual data points are displayed to enhance clarity.

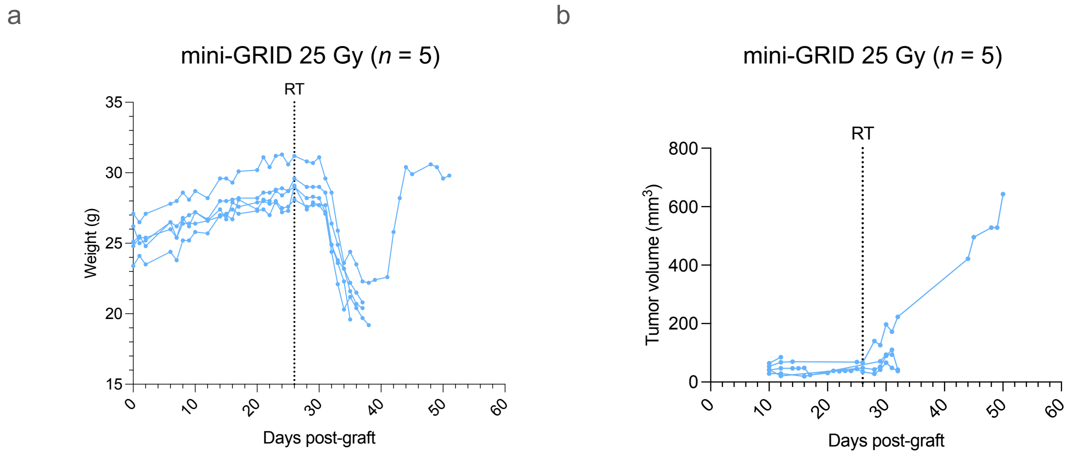


**Figure S2. Individual weight and tumor volume curves in the mini-GRID 25 Gy group.** Data from two independent experiments. Individual data points are displayed to enhance clarity.

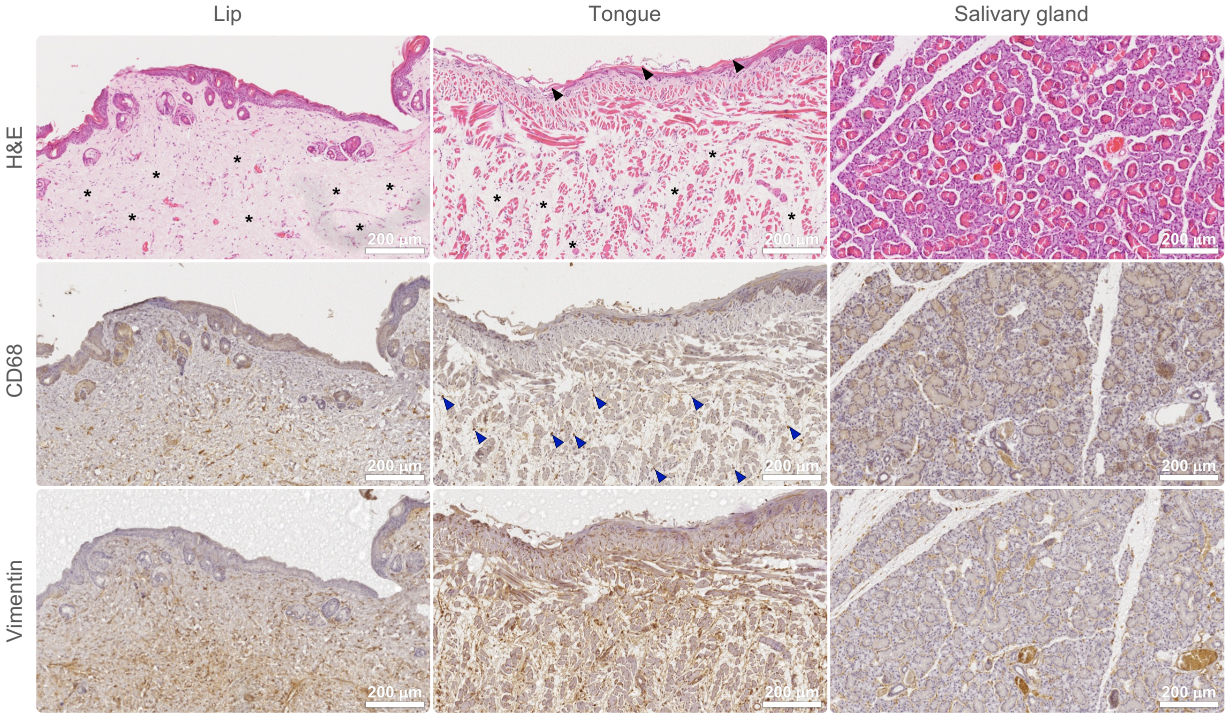


**Figure S3. Histological analysis of healthy tissues following mini-GRID RT at 25 Gy.** H&E staining, as well as CD68 and vimentin immunostaining, were performed on sections of lip skin (*n* = 4), tongue (*n* = 4), and submandibular salivary glands (*n* = 4) collected from a subset of mice in the mini-GRID 25 Gy group. Black arrowhead: loss of filiform papillae; black dashed area: increased blood vessel size; blue arrowhead: monocyte/macrophage infiltration.

| Days | Control group (*n* = 6) | Conventional RT 20 Gy (*n* = 7) | Mini-GRID RT 20 Gy (*n* = 7) |
|---|---|---|---|
| 0 | 6 | 7 | 7 |
| 24 | 6 | | |
| 29 | 5 | 7 | |
| 34 | | 6 | 7 |
| 35 | 4 | 5 | |
| 36 | | 3 | 6 |
| 37 | 3 | | |
| 38 | | | 5 |
| 39 | | 1 | |
| 43 | 2 | | |
| 45 | 1 | | |
| 56 | | | 4 |
| 57 | | | 2 |
| 58 | | | 1 |

**Table S1. Effectives at risk in the survival study.**

| | | | | | *Cause of sacrifice* | | |
|---|---|---|---|---|---|---|---|
| **Protocol** | **n** | **Surviving mice** | **Median survival (days)** | **Signs of pain** | **Weight loss > 20% highest weight** | **Tumor size > 600 mm$^3$** | **Pain** |
| Mini-GRID 25 Gy | 5 | 0 | 37 | 3 | 3 | 1 | 1 |

**Table S2. Median survival and causes of sacrifice in the mini-GRID 25 Gy group.**
